# Developing a Nomogram for Prioritizing Hysteroscopy in Endometrial Cancer Diagnosis: A Case-Control Study

**DOI:** 10.3390/jcm13041145

**Published:** 2024-02-18

**Authors:** Bruna Bottura, Raphael Federicci Haddad, Vanessa Alvarenga-Bezerra, Vinicius Campos, Luiza Perez, Carolina Resende, Fernanda de Almeida Asencio, Adolfo Wenjaw Liao, Mariano Tamura Vieira Gomes, Eduardo Zlotnik, Renato Moretti-Marques

**Affiliations:** 1 Ginecologia Oncológica, Hospital Municipal da Vila Santa Catarina Dr. Gilson de Cássia Marques de Carvalho, São Paulo 04378-500, SP, Brazil; brunabottura_26@hotmail.com (B.B.); vanessa.abezerra@einstein.br (V.A.-B.); renato.moretti@einstein.br (R.M.-M.); 2 Programa de Saúde da Mulher, Hospital Israelita Albert Einstein, São Paulo 05652-900, SP, Brazil; vinicius.moreira.campos@gmail.com (V.C.); fedealmeida1982@hotmail.com (F.d.A.A.); adolfo.liao@gmail.com (A.W.L.); zlotnik@einstein.br (E.Z.); 3 Weill Cornell Medicine, New York, NY 10075, USA; lrp4001@med.cornell.edu

**Keywords:** endometrial cancer, hysteroscopy, COVID-19, risk analyses, gynecologic surgical procedures, Brazil, health, health system, resource allocation

## Abstract

(1) Background: The pandemic led to significant healthcare disruptions, resulting in postponed surgeries and extended waiting times for non-urgent treatments, including hysteroscopies essential for diagnosing endometrial cancer. This study aims to formulate a risk stratification model to enhance the prioritization of hysteroscopy procedures in Brazil; (2) Methods: A case-control study was conducted at Vila Santa Catarina Hospital in São Paulo, analyzing the medical records of 2103 women who underwent hysteroscopy between March 2019 and March 2022. We used bivariate analysis and multivariate linear regression to identify risk factors associated with endometrial cancer and formulate a nomogram; (3) Results: The findings revealed a 5.5% incidence of pre-invasive and invasive endometrial disease in the study population, with an average waiting time of 120 days for hysteroscopy procedures. The main risk factors identified were hypertension, diabetes, postmenopausal bleeding, and obesity; (4) Conclusions: This research highlights the urgent need for efficient prioritization of hysteroscopy procedures in the wake of the pandemic. The developed nomogram is an innovative tool for identifying patients at higher risk of endometrial cancer, thus facilitating timely diagnosis and treatment and improving overall patient outcomes in a strained healthcare system.

## 1. Introduction

The COVID-19 pandemic is a global health crisis that has negatively impacted the organization and infrastructure of public health, resulting in a significant number of deaths and disease-related morbidity [1,2,3]. Globally, it is estimated that over 7.2 trillion dollars have been spent by May 2022 to contain its spread and consequences [1,2,3]. Approximately 28 million surgeries are believed to have been postponed or canceled [1,2]. In the United Kingdom, the waiting list for non-urgent treatments reached a record high, jumping from 4.4 million in 2019 to 5.6 million in 2021 due to the pandemic [1,2,3]. In Brazil, a similar situation was observed with a 41.5% reduction in elective surgeries [1,2]. The exacerbation of pre-existing conditions, worsened prognosis, morbidity, decreased functional capacity of the population, and the subsequent negative financial impact on health and social security systems are estimated consequences [1,2].

GLOBOCAN estimated around 18 million cases of malignant diseases in 2020 [4]. However, with the pandemic, a decline of 40% to 76% in the diagnosis and treatment of various cancers is expected [1,2,3]. Endometrial cancer is one of the most common cancers among women, with an estimated lifetime risk of 5%, and the second most lethal in developed countries [4]. The prognosis for this disease depends on factors such as clinical performance, histopathological type, stage, and treatment received [4]. The time interval between the symptom onset and the treatment initiation is correlated with survival and cost [5,6,7].

The diagnosis of endometrial pre-malignant and malignant conditions often involves endometrial sampling and imaging techniques. Hysteroscopy, enables visualizations the uterine cavity [8,9], and thus, evaluation of abnormal uterine bleeding and intrauterine lesions [10]. Additionally, hysteroscopy is considered the gold standard for evaluating intracavitary pathology in patients with abnormal uterine bleeding and in those suspected of having cavity abnormalities in infertility cases [8]. Office-based operative hysteroscopy has revolutionized clinical practice with its “see-and-treat” modality, allowing patients to resume activities immediately and avoiding the risks associated with anesthesia and operating room procedures [8,10,11].

In Brazil’s Unified Health System (SUS), the referral process for specialized procedures, such as hysteroscopy, begins at the Basic Health Units (UBS) [12]. At these units, general practitioners assess patients’ symptoms and, if they identify a need for more complex examinations, they refer patients to specialized services. This referral system ensures that patients are directed to centers with the necessary resources and expertise to perform procedures like hysteroscopy, thus maintaining continuity of medical care within the public health network [12,13].

The structural limitations of hysteroscopy services predated the COVID-19 pandemic, given the high demand for this procedure [13,14,15,16]. The mobility restrictions, shortage of healthcare resources, and the population’s fear of contracting COVID-19 exacerbated an already strained system. As a result, there was a 20% reduction in the rate of endometrial cancer diagnosis and a 43% reduction in the diagnosis of precursor lesions in 2020 [2,3,15]. There was also a 33% reduction in complaints of postmenopausal uterine bleeding in gynecological consultations during this period [17,18].

With easing restrictions and the return of population mobility, long waiting lists for hysteroscopy procedures are observed, both for women with and without endometrial cancer [3,14,15]. Therefore, this pioneering study aims to create a risk stratification model for the prediction of endometrial cancer and its precursor lesions among individuals on the waiting list for hysteroscopy examinations. The findings can inform healthcare policy, especially in optimizing the allocation of resources and managing patient queues effectively in gynecology departments.

## 2. Materials and Methods

### 2.1. Patients

A case-control study was conducted among patients with suspected endometrial cancer, including 2103 women who underwent hysteroscopy at Vila Santa Catarina Hospital-Society, Israelita Albert Einstein, in São Paulo, Brazil, between March 2019 and March 2022. One hundred and fifty-eight patients were excluded due to incorrect indication or unnecessary procedure. The cases were considered patients with endometrial cancer proven histologically in a hysteroscopic endometrial biopsy, and the controls were those that underwent biopsy hysteroscopy and were not diagnosed with endometrial cancer. Retrospective medical record data extraction included demographic information, obstetric history, medical comorbidities, obesity, tobacco use, tamoxifen use, menopause, postmenopausal bleeding, hormone replacement therapy, and ultrasound findings.

### 2.2. Data Collection and Variables

Variables collected included demographic details, medical history, clinical findings, results from transvaginal ultrasounds, hysteroscopic observations, and histopathological outcomes. The variables were categorized and coded for ease of analysis, with continuous variables such as age, body mass index (BMI), and endometrial thickness and categorical variables including hypertension, diabetes, and postmenopausal bleeding status. All data handling processes were designed to ensure confidentiality and compliance with data protection regulations.

### 2.3. Hysteroscopy Procedure

The hysteroscopic evaluations were conducted in an ambulatory setting at Vila Santa Catarina Hospital-Society, Israelita Albert Einstein. These procedures were performed by medical staff and medical residents under attending supervision, ensuring a high standard of care and consistency in technique despite operator variability. While providing educational training to residents, this structured approach to the hysteroscopies maintained procedural uniformity across the study, contributing to the reliability of the clinical findings.

### 2.4. Statistical Analysis

Initially, the study employed descriptive statistical methods to summarize the data. This involved calculating means and standard deviations for continuous variables (e.g., age, BMI, endometrial thickness). For categorical variables (e.g., presence of hypertension, diabetes, history of postmenopausal bleeding), frequency distributions were utilized to determine their prevalence in the study population. The Shapiro-Wilk test was applied to assess the normality of the distributions of continuous variables, ensuring the appropriate use of statistical tests and models.

Comparative statistical tests were employed to explore the relationships between various risk factors and the presence of premalignant or malignant lesions. The Mann-Whitney U test was employed to compare nonparametric continuous variables, and the Chi-squared or Fisher’s exact tests were used for categorical data comparisons.

Multivariate analysis was conducted using Generalized Linear Models (GLM), incorporating variables with a significance level of up to 20% from bivariate analyses. Multicollinearity was assessed and addressed before the final model selection through a backward elimination process, using Wald test. The Random Over Sampling Examples (ROSE) method was used to balance the data set, addressing the class imbalance prevalent in the outcome variable.

The final model’s predictive power was evaluated using receiver operating characteristic (ROC) curve analysis. This helped identify an optimal cutoff value for predicting the risk of lesions. The nomogram function on R was used to create a linear predictor function of the risk of endometrial cancer based on the final regression model. Scores for each variable were calculated to determine their contribution to the overall endometrial cancer risk. All tests were two-tailed, and a *p*-value of less than 0.05 was considered statistically significant, indicating that the findings were unlikely due to chance. A significance level of 5% was adopted for all tests. All analyses were performed using R software, version 4.1.1.

## 3. Results

**Figure 1:**
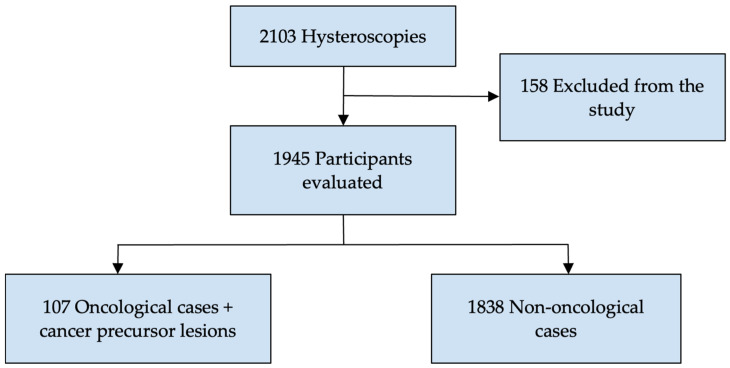
Study participant case flowchart.

While 2103 women were eligible for the study, 158 were excluded because the hysteroscopy was not completed due to the wrong procedure indication. Therefore, 1945 participants were included in the final analysis. Among them, 107 cases were diagnosed with pre-invasive or invasive endometrial cancer (Figure 1).

Table 1 and Table 2 present the demographic and clinical characteristics of the study participants in categorical and numerical formats, respectively. Since the data did not follow a normal distribution, medians and interquartile ranges (IQR) were employed for more accurate representation.

Hypertension was the most common comorbidity, affecting approximately 39.59% of participants, followed by diabetes (17.33%), hypothyroidism (10.44%), and dyslipidemia (13.52%). Postmenopausal bleeding was reported in nearly a third of the case group, with 6.63% on hormone replacement therapy and 1.44% on tamoxifen. Ultrasound findings included polyps (18.62%), adenomyosis (0.21%), and leiomyomas (14.46%). The median uterine volume was 78 cc, with a median endometrial thickness of 7 mm.

Table 3 describes the study sample stratified by group: cancer or precursor lesion and control group and the comparison between the outcome variable and each risk factor considered.

The significant independent variables were: Hypertension (*p* < 0.001), diabetes (*p* < 0.001), current stage (*p* < 0.001), post-menopausal bleeding (*p* < 0.001), ultrasound findings: adenomyosis (*p* < 0.001), ultrasound findings: polyp (*p* 0.002), and duration of menopause (*p* < 0.001). All variables listed above were included in multivariate analysis except adenomyosis on ultrasound due its low prevalence.

The proportion of postmenopausal bleeding was significantly higher in the case group (*p* < 0.001). While the use of hormonal therapy and Tamoxifen did not show a significant difference, the presence of adenomyosis was significantly associated with the case group. No significant associations were observed with leiomyoma or polyp presence.

Table 4 shows the distribution of histologic types among endometrial cancer cases diagnosed during hysteroscopy. The most common type was low-grade endometrioid carcinoma [FIGO G1/G2], corresponding to 47.6% of the cases, followed by endometrial hyperplasia with and without atypia, representing 24.2% of cases. One case of bladder cancer and four cases of cervical cancer were diagnosed.

### Modeling

During the modeling phase, certain variables previously discarded due to lack of significance in bivariate analysis or a high number of missing values were re-included due to their recognized importance as risk factors. This included variables such as endometrial thickness.

Multicollinearity was assessed, particularly for the variable “number of pregnancies”, which exhibited significant multicollinearity with “vaginal deliveries”, “cesarean sections”, and “spontaneous abortions”. Hence, these variables were excluded when ‘number of pregnancies’ was included in the model. Table 5 details the Variance Inflation Factor (VIF) values for these variables. Furthermore, given the understanding of the overlap between age and the duration of menopause, we opted to include only age in the final modeling.

Table 6 presents the results of the multivariate linear regression between endometrial cancer and precursor lesions and the following dependent variables: hypertension, diabetes, post-menopausal bleeding, endometrial polyp, age, uterine volume, number of pregnancies, BMI, and endometrial thickness. All variables had statistically significant associations with the diagnosis of endometrial cancer or pre-invasive lesions in bivariate analysis.

The absence of high blood pressure, diabetes, and postmenopausal bleeding were associated with a lower risk of endometrial cancer by 22.02%, 49.4%, and 55.51%, respectively. A higher number of pregnancies also acted as a protective factor. For each additional pregnancy, there was a 12.81% reduction in endometrial cancer risk. An increase in BMI was associated with a 3.60% heightened risk per unit [Kg/m^2^], and each additional year of age contributed to a 3.62% increase in risk. Similarly, a rise in uterine volume and endometrial thickness corresponded to increased risks of 0.27% and 3.84% per unit increase, respectively. Additionally, the presence of endometrial polyps was linked to a 37.57% higher chance of cancer.

**Figure 2:**
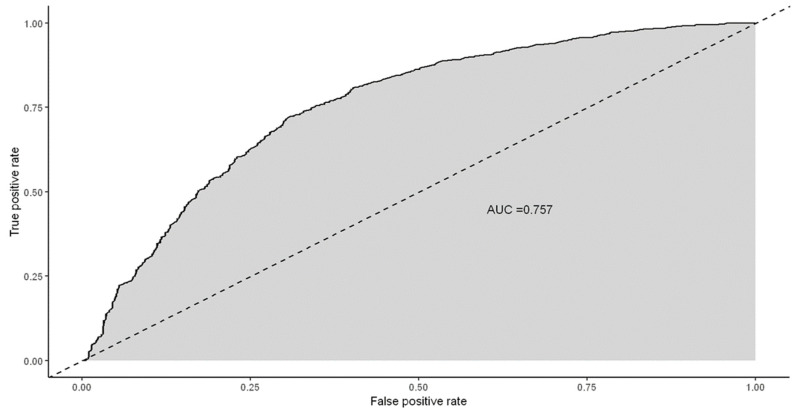
ROC curve of the final multivariate logistic regression.

Based on the statistically significant risk factors for the diagnosis of invasive and pre-invasive endometrial disease, a ROC curve was calculated for the logistic regression model. Figure 2 illustrates the area under the curve (AUC) of 75.7%. Two cutoff points, 0.5 and 0.35, were considered to assess the model’s effectiveness. For a cutoff point set at 0.5, the model showed an accuracy of 70.3% and a sensitivity of 64.7%. When adjusting the cutoff point to 0.35, there was a noticeable improvement in sensitivity, reaching 83.0%, although specificity and accuracy slightly decreased to 49.5% and 65.6%, respectively.

Supplementary Materials Table S1 depicts the nomogram derived from the logistic regression model to stratify the risk of endometrial cancer and precursor lesion diagnosis. It illustrates the characteristics of the participants that contribute to the final score and their corresponding risk of diagnosis.

## 4. Discussion

Identifying malignant lesions based solely on endometrial thickness presents significant challenges. Transvaginal ultrasound (TVUS) is a non-invasive alternative to endometrial sampling, yet its efficacy in detecting endometrial carcinoma varies [16,17,18]. Studies have shown high sensitivity at certain thickness thresholds, but others indicate limited reliability for excluding cancer based solely on this measure [16,18,19,20]. The observed referral pattern, predominantly due to endometrial thickening, may have contributed to the non-significant difference in endometrial thickness between groups. This underscores the challenges in differentiating between benign and malignant cases and the importance of effective triage systems.

The methods for diagnosing endometrial cancer are well-known [8,13,21,22]. Usually, patients are diagnosed at an early stage, leading to a good prognosis [21,23]. However, this is not as common in Brazil, and more advanced stages are frequently encountered [23]. In our service, we have extensive experience with diagnostic hysteroscopy, the chosen procedure for endometrial evaluation. This practice is supported by current guidelines, which advocate for hysteroscopy as the most accurate and cost-effective targeted biopsy method for diagnosing endometrial pathologies, particularly malignancies [10,24]. According to an evidence-based guideline for clinical practice, hysteroscopy surpasses blind methods in diagnostic accuracy and should be the first choice in patients with suspected endometrial cancer.

Worldwide, the diagnosis of cancer was postponed during and after the COVID-19 pandemic, and endometrial cancer was no exception [4,25,26]. Despite the low malignancy rate among patients undergoing hysteroscopy, long waiting lists for the procedure are observed [18]. The high number of hysteroscopy indications, coupled with limitations in human and financial resources, and the poor organizational structure of the healthcare service, which were exacerbated by the COVID-19 pandemic, resulted in longer waiting times for the procedure [26]. Therefore, it is necessary to identify patients on the waiting list with a higher risk of endometrial cancer or precursor lesion diagnosis and prioritize them.

This study arises from the realization of the long waiting list for hysteroscopy, which has become even more prolonged due to the COVID-19 pandemic, and the lack of scientific evidence to support the prioritization of patients based on the risk of having endometrial cancer or precursor lesion diagnosis. As a result, we created a risk stratification model for the diagnosis of invasive and pre-invasive endometrial lesions based on the individual’s risk factors and correlating them with their respective clinical and histological diagnostic data.

The core idea was to devise a strategy to prioritize patient queues in referral centers after initial assessments by general practitioners in the primary care setting. This strategy is particularly pertinent within the SUS, where patients often face long wait times for specialized procedures. By implementing a nomogram-based model for patient prioritization, the study aims to streamline the process, allowing quicker intervention for those at higher risk and potentially improving outcomes for patients with endometrial pathologies.

A similar situation is observed in other areas, such as liver transplantation and managing patients with sepsis [27,28]. For liver transplantation, predefined values of creatinine, bilirubin, international normalized ratio (INR), the presence or absence of cancer, and the need for dialysis are considered to classify patients into subcategories to determine the prioritization of liver transplantation, called the Model for End-Stage Liver Disease (MELD) [27]. In the diagnosis of sepsis, patient triage is based on the assessment of the respiratory system, blood pressure, and neurological system to prioritize care, using the Quick Sequential Sepsis-related Organ Failure Assessment (qSOFA) score [28].

Subsequent studies can validate the risk subcategorization strategy for endometrial cancer among individuals on the hysteroscopy waiting list, aiming to prioritize examinations with a higher probability of malignancy. The development of an application, similar to the International Ovarian Tumor Analysis (IOTA) ADNEX for adnexal masses, to assist in the referral and prioritization for the hysteroscopy waiting list is envisioned [29]. Once validated, the risk stratification could be implemented by government initiatives to optimize hysteroscopy queues and mitigate the advanced stages of endometrial cancer resulting from delayed diagnosis.

In addition, we propose drawing a parallel with the Breast Imaging Reporting and Data System (BI-RADS) to enhance result interpretation and decision-making when prioritizing the procedure [30]. By comparing our nomogram with the BI-RADS system, we can establish a clear parallel and create a more objective classification system, which we term the Endometrial Malignancy Prediction System (EMPS), as detailed in Table 7:

- A score under 70 on the nomogram corresponds to EMPS 1 (very low risk), where the risk of endometrial cancer or precursor lesions is extremely low (<5%).
- Scores between 70 and 88 on the nomogram align with EMPS 2 (low risk), where the risk is less than 10%.
- Between 89 and 143, we find a transition zone covering EMPS 3 (medium risk) with a risk of endometrial cancer or precursor lesions between 10% and 50%. EMPS 4 (high risk), with scores ranging from 144 to 197, encompasses a wide range of risks, from 50% to 90%.
- Scores above 197 on the nomogram correlate with EMPS 5 (very high risk), indicating a risk of endometrial cancer or precursor lesions exceeding 90%, which should be prioritized.

Incorporating a risk stratification approach for surgical queue prioritization in Brazil’s public health system, SUS, is crucial due to the country’s vast geographical size and diverse healthcare settings. Given varying access to medical technology across Brazil, a straightforward and practical tool aligns with the realities of SUS [31]. This approach facilitates equitable resource distribution, particularly in under-resourced areas, enabling more informed decision-making by healthcare providers, aiming to reduce wait times for essential procedures, and improving patient outcomes in a system with notable disparities in care access and quality.

This study, although offering a valuable tool in the form of the nomogram, has inherent limitations related to its design and context. Designed to be applicable in low resource settings, the decision to use a nomogram was made due to its ease of application, even though other methodologies, such as machine learning techniques, exist for estimating risk based on data. Furthermore, not testing the algorithm on separate samples from the development dataset underscores the need for future external validations. The oversampling method, adopted due to the scarcity of malignant cases, may have introduced biases through the generation of synthetic data. Additionally, this study emphasizes the absence of standardization and the need for improved patient selection criteria in the referral process for hysteroscopy, placing our nomogram in a broader diagnostic context.

Finally, since the focus was on patients within the Brazilian Unified Health System (SUS), many of whom face challenges in accessing timely healthcare, it may limit the generalizability of the findings to other groups or contexts.

## 5. Conclusions

This pioneering study introduces a new tool for predicting the risk of endometrial premalignant or malignant lesions in patients on the hysteroscopy waiting list, to improve the management of the waiting queue for this procedure. As a result, patients needing prompt attention can have their procedures prioritized. Out of the cases, 5.5% were in this group and were in the same 120-day waiting queue for the examination, a timeframe that can significantly impact disease survival. Further studies are needed to validate this nomogram and assess its effectiveness in organizing hysteroscopy services. They should compare the effectiveness of this nomogram-based approach with traditional methods of patient prioritization for hysteroscopy.

## Figures and Tables

**Table 1:** Characterization of the study sample (categorical variable).

Variable	Classes	Nº (%)
Hypertension		
	Yes	770 (39.59)
	No	1175 (60.41)
Diabetes		
	Yes	337 (17.33)
	No	1608 (82.67)
Hypothyroidism		
	Yes	203 (10.44)
	No	1742 (89.56)
Dyslipidemia		
	Yes	263 (13.52)
	No	1682 (86.48)
Obesity		
NA = 1724	Yes	221 (100)
Sd ischemic		
	Yes	42 (2.16)
	No	1903 (97.84)
Smoking		
NA = 1918	Yes	27 (100)
Tamoxifen use		
	Yes	28 (1.44)
	No	1917 (98.56)
Current phase		
	Menopause	1237 (63.6)
	Management	708 (36.4)
Bleeding after menopause		
	Yes	379 (19.49)
	No	1566 (80.51)
Hormonal method		
	Yes	129 (6.63)
	No	1816 (93.37)
US findings: myoma		
NA = 1	Yes	280 (14.4)
	No	1664 (85.6)
Us Findings: Polipo		
NA = 1	Yes	362 (18.62)
	No	1582 (81.38)
US findings: adenomyosis		
NA = 1	Yes	4 (0.21)
	No	1940 (99.79)
Group		
NA = 4	Cancer	107 (5.51)
	Control	1834 (94.49)

NA = not available.

**Table 2:** Characterization of the study sample (continuous variable).

Variable (N)	Median (IQR)
Age (1872)	57 (47–64)
Age at menopause (1189)	50 (47–52)
Duration of menopause (1173)	12 (6–19)
Number of pregnancies (1896)	3 (2–4)
Number of vaginal births (1858)	1 (0–3)
No. of C-sections (1810)	0 (0–1)
Number of spontaneous abortions (1790)	0 (0–1)
Number of induced abortions (1288)	0 (0–0)
Uterine volume (cc) (1853)	78 (46.7–137.5)
BMI (473)	28.69 (25.1–33.66)
Endometrial thickness (mm) (1805)	7 (4–10)

IQR: interquartile range.

**Table 3:** Characterization of the Study Sample by group.

		Group		
Variable	Class (n)	Cancer or Precursor Lesion	Control	*p*-Values
		N/Median	N/Median	
Age		62 (58–69)	56 (46–64)	<0.001
Age at menopause		50 (45–53)	50 (47–52)	0.698
Duration of menopause		15 (9.25–20.75)	12 (6–18)	0.002
Number of pregnancies		2 (2–4)	3 (2–4)	0.434
Number of vaginal births		2 (0–3)	1 (0–3)	0.143
Number of C-sections		0 (0–1)	1 (0–1)	0.009
Number of miscarriages		0 (0–0)	0 (0–1)	0.061
Uterine volume (cc)		96.9 (57.8–173.6)	77.2 (46–134.5)	0.005
BMI		32.85 (27.85–39.97)	28.52 (25–33.2)	0.04
Endometrial thickness (mm)		7.7 (3–12)	7 (4–10)	0.515
Hypertension	Yes (768)	61 (57.01)	707 (38.55)	<0.001
Diabetes	Yes (334)	41 (38.32)	293 (15.98)	<0.001
Hypothyroidism	Yes (202)	13 (12.15)	189 (10.31)	0.704
Hyperlipidemia	Yes (262)	17 (15.89)	245 (13.36)	0.695
Ischemic heart disease	Yes (41)	3 (2.8)	38 (2.07)	0.804
Tamoxifen use	Yes (28)	2 (1.87)	26 (1.42)	0.999
Menopause Status	Post-menopausal (1234)	94 (87.85)	1140 (62.16)	<0.001
	Premenopausal (707)	13 (12.15)	694 (37.84)	
Post-menopausal bleeding	Yes (377)	66 (61.68)	311 (16.96)	<0.001
Hormonal therapy	Yes (128)	2 (1.87)	126 (6.87)	0.132
Ultrasound findings: leiomyoma	Yes (279)	11 (10.28)	268 (14.61)	0.327
Ultrasound findings: polyp	Yes (361)	7 (6.54)	354 (19.3)	0.002
Ultrasound findings: adenomyosis	Yes (4)	4 (3.74)	0 (0)	<0.001

Chi-squared test or Fisher Exact Test (5% significance level) for qualitative variables/Mann-Whitney Test (5% significance level) for quantitative variables. Values in parentheses as interquartile range, or percentage.

**Table 4:** Histologic findings of patients with hysteroscopic biopsy abnormalities.

Histologic Type	Number of Cases	Percentage
Low-grade endometrial carcinoma (Grade 1 and 2)	51	47.60%
Poorly differentiated carcinomas (endometrioid, serous, undifferentiated)	20	18.60%
Endometrial hyperplasia with or without atypia	26	24.2%
Uterine sarcoma	5	4.60%
Other malignancies (bladder. cervix)	5	4.6%

**Table 5:** Multicollinearity Analysis.

Variable (VIF)	VIF—Complete	VIF—No Number of Pregnancies
Age	2.95	2.98
Duration of menopause	2.76	2.80
Vaginal births	86,482,820	1.37
Cesarean sections	18,140,880	1.35
Miscarriages	11,035,760	1.05
Endometrial thickness	1.17	1.17
Uterine volume (cc)	1.05	1.05
Number of pregnancies	93,858,950	-
BMI	1.3	1.26

VIF: Variance inflation factor.

**Table 6:** Multivariate regression of endometrial cancer or precursor lesions and risk factors.

Variables	Estimates	Odds Ratio	Confidence Interval (95%)	*p*-Value
Hypertension (No)	−0.249	0.780	(0.62–0.98)	0.033
Diabetes (No)	−0.682	0.506	(0.408–0.626)	<0.001
Postmenopausal bleeding (No)	−0.810	0.445	(0.36–0.549)	<0.001
Endometrial polyp (Yes)	0.319	1.376	(1.010–1.873)	0.042
Age (year)	0.036	1.036	(1.024–1.049)	<0.001
Uterine volume (cc)	0.003	1.003	(1.002–1.003)	<0.001
Number of pregnancies	−0.137	0.872	(0.835–0.91)	<0.001
BMI (Kg/m^2^)	0.035	1.036	(1.025–1.047)	<0.001
Endometrial thickness (mm)	0.038	1.038	(1.02–1.057)	<0.001

Wald’s Test (5% significance level).cc: cubic centimeter; mm: millimeter.

**Table 7:** Endometrial Malignancy Prediction System Classification.

EMPS	Nomogram Score	Cancer or Precursor Lesion Risk
1—Very low risk	<70	<5%
2—Low risk	70–88	5–10%
3—Medium risk	89–143	10–50%
4—High risk	144–197	50–90%
5—Very high risk	>197	>90%

EMPS: Endometrial Malignancy Prediction System.

## Data Availability

If anyone wishes to access the dataset used in our study, please contact the authors directly to request the data.

## Supplementary Materials

The following supporting information can be downloaded at: https://www.mdpi.com/article/10.3390/jcm13041145/s1, Table S1: Scoring tables to obtain endometrial cancer and precursor lesion risk (Supplement).

## References

[1] Coronavírus: Os 10 Países que mais Gastaram para Enfrentar a Pandemia de Covid-19—BBC News Brasil. https://www.bbc.com/portuguese/internacional-52721417.

[2] COVIDSurg Collaborative (2020). Elective surgery cancellations due to the COVID-19 pandemic: Global predictive modelling to inform surgical recovery plans. Br. J. Surg..

[3] Surgeons Call for a ‘New Deal for Surgery’ to Reduce the ‘Colossal’ Elective Backlog—Royal College of Surgeons. https://www.rcseng.ac.uk/news-and-events/media-centre/press-releases/new-deal-for-surgery-2021/.

[4] Sung H., Ferlay J., Siegel R.L., Laversanne M., Soerjomataram I., Jemal A., Bray F. (2021). Global cancer statistics 2020: GLOBOCAN estimates of incidence and mortality worldwide for 36 cancers in 185 countries. CA Cancer J. Clin..

[5] Candido E.C., Veiga Junior N.N., Minari M.P., Toledo M.C.S., Yela D.A., Teixeira J.C. (2021). Malignant Uterine Neoplasms Attended at a Brazilian Regional Hospital: 16-years Profile and Time Elapsed for Diagnosis and Treatment. Rev. Bras. Ginecol. Obstet..

[6] Weiderpass E., Antoine J., Bray F.I., Oh J.-K., Arbyn M. (2014). Trends in corpus uteri cancer mortality in member states of the European Union. Eur. J. Cancer..

[7] Uterine Cancer Statistics Cancer Research UK. https://www.cancerresearchuk.org/health-professional/cancer-statistics/statistics-by-cancer-type/uterine-cancer.

[8] Salazar C.A., Isaacson K.B. (2018). Office operative hysteroscopy: An update. J. Minim. Invasive Gynecol..

[9] American College of Obstetricians and Gynecologists (2020). The use of hysteroscopy for the diagnosis and treatment of intrauterine pathology: ACOG committee opinion, number 800. Obstet. Gynecol..

[10] D’Urso V., Gulino F.A., Incognito G.G., Cimino M., Dilisi V., Di Stefano A., Gulisano M., Cannone F., Capriglione S., Palumbo M. (2023). Hysteroscopic findings and operative treatment: All at once?. J. Clin. Med..

[11] Orlando M.S., Bradley L.D. (2022). Implementation of office hysteroscopy for the evaluation and treatment of intrauterine pathology. Obstet. Gynecol..

[12] Wahhab Kucharski K., Battisti I.D.E., Fernandes D.M.M., Anastácio Z.F.C. (2022). Políticas Públicas de Saúde no Brasil: Uma Trajetória do Império a Criação do Sus. Revista Contexto & Educação.

[13] Machado C.V., Silva G.A.E. (2019). Political struggles for a universal health system in Brazil: Successes and limits in the reduction of inequalities. Glob. Health.

[14] Rathnayake D., Clarke M. (2021). The effectiveness of different patient referral systems to shorten waiting times for elective surgeries: Systematic review. BMC Health Serv. Res..

[15] Goldstein R.B., Bree R.L., Benson C.B., Benacerraf B.R., Bloss J.D., Carlos R., Fleischer A.C., Goldstein S.R., Hunt R.B., Kurman R.J. (2002). Evaluation of the woman with postmenopausal bleeding. J. Ultrasound Med..

[16] Tabor A., Watt H.C., Wald N.J. (2002). Endometrial thickness as a test for endometrial cancer in women with postmenopausal vaginal bleeding. Obstet. Gynecol..

[17] Smith-Bindman R., Weiss E., Feldstein V. (2004). How thick is too thick? When endometrial thickness should prompt biopsy in postmenopausal women without vaginal bleeding. Ultrasound Obstet. Gynecol..

[18] Zhang L., Guo Y., Qian G., Su T., Xu H. (2022). Value of endometrial thickness for the detection of endometrial cancer and atypical hyperplasia in asymptomatic postmenopausal women. BMC Womens Health..

[19] Yerrisani J., Kothari A., Collins K., Ballard E., Kothari A. (2022). Evaluation of endometrial thickness by transvaginal ultrasound and baseline risk factors as a predictor for endometrial abnormalities in postmenopausal women. Australas. J. Ultrasound Med..

[20] Breijer M.C., Peeters J.A.H., Opmeer B.C., Clark T.J., Verheijen R.H.M., Mol B.W.J., Timmermans A. (2012). Capacity of endometrial thickness measurement to diagnose endometrial carcinoma in asymptomatic postmenopausal women: A systematic review and meta-analysis. Ultrasound Obstet. Gynecol..

[21] Clarke M.A., Long B.J., Del Mar Morillo A., Arbyn M., Bakkum-Gamez J.N., Wentzensen N. (2018). Association of Endometrial Cancer Risk with Postmenopausal Bleeding in Women: A Systematic Review and Meta-analysis. JAMA Intern. Med..

[22] American College of Obstetricians and Gynecologists (2009). ACOG Committee Opinion No. 426: The role of transvaginal ultrasonography in the evaluation of postmenopausal bleeding. Obstet. Gynecol..

[23] Ferlay J., Soerjomataram I., Dikshit R., Eser S., Mathers C., Rebelo M., Parkin D.M., Forman D., Bray F. (2015). Cancer incidence and mortality worldwide: Sources, methods and major patterns in GLOBOCAN 2012. Int. J. Cancer.

[24] Vitale S.G., Buzzaccarini G., Riemma G., Pacheco L.A., Di Spiezio Sardo A., Carugno J., Chiantera V., Török P., Noventa M., Haimovich S. (2023). Endometrial biopsy: Indications, techniques and recommendations. An evidence-based guideline for clinical practice. J. Gynecol. Obstet. Hum. Reprod..

[25] Kaufman H.W., Chen Z., Niles J., Fesko Y. (2020). Changes in the Number of US Patients with Newly Identified Cancer Before and during the Coronavirus Disease 2019 (COVID-19) Pandemic. JAMA Netw. Open.

[26] Suh-Burgmann E.J., Alavi M., Schmittdiel J. (2020). Endometrial Cancer Detection during the Coronavirus Disease 2019 (COVID-19) Pandemic. Obstet. Gynecol..

[27] Kim W.R., Mannalithara A., Heimbach J.K., Kamath P.S., Asrani S.K., Biggins S.W., Wood N.L., Gentry S.E., Kwong A.J. (2021). MELD 3.0: The Model for End-Stage Liver Disease Updated for the Modern Era. Gastroenterology.

[28] Qiu X., Lei Y.-P., Zhou R.-X. (2023). SIRS, SOFA, qSOFA, and NEWS in the diagnosis of sepsis and prediction of adverse outcomes: A systematic review and meta-analysis. Expert Rev. Anti-Infect. Ther..

[29] Hiett A.K., Sonek J.D., Guy M., Reid T.J. (2022). Performance of IOTA Simple Rules, Simple Rules risk assessment, ADNEX model and O-RADS in differentiating between benign and malignant adnexal lesions in North American women. Ultrasound Obstet. Gynecol..

[30] Balleyguier C., Ayadi S., Van Nguyen K., Vanel D., Dromain C., Sigal R. (2007). BIRADS classification in mammography. Eur. J. Radiol..

[31] Fernandes F.M.B. (2017). Regionalization in the Brazilian Healthcare System, SUS: A critical review. Cien Saude Colet..

