# Supplementary material for: Developing a Nomogram for Prioritizing Hysteroscopy in Endometrial Cancer Diagnosis: A Case-Control Study
Source: J Clin Med. 2024 Feb 18;13(4):1145. doi: 10.3390/jcm13041145 (PMC10889308; doi:10.3390/jcm13041145)
Supplement: Supplementary file 1 [file jcm-13-01145-s001.zip › jcm-2823678-supplementary.pdf]

Supplementary Table S1. Scoring tables to obtain endometrial cancer and precursor lesion risk (Supplement)

Hypertension	Score
Yes	6
No	0

Diabetes	Score
Yes	17
No	0

Postmenopausal bleeding	Score
Yes	20
No	0

Uterus volume	Score
20	0
120	7
220	13
320	20
420	27

Age	Score	Endometrial thickness	Score
18	0	1	0
20	2	2	1
22	4	3	2
24	5	4	3
26	7	5	4
28	9	6	5
30	11	7	6
32	12	8	7
34	14	9	7
36	16	10	8
38	18	11	9
40	19	12	10
42	21	13	11
44	23	14	12
46	25	15	13
48	26	16	14
50	28	17	15

Total	Endometrial cancer and precursor lesion risk
70	0,05
88	0,10
109	0,20
122	0,30
133	0,40
143	0,50
153	0,60
164	0,70
177	0,80
197	0,90
216	0,95

520	33
620	40
720	47
820	53
920	60
1020	67
1120	73
1220	80
1320	87
1420	93
1520	100

Pregnancy	Score
0	61
2	54
4	48
6	41
8	34
10	27
12	20
14	14
16	7

52	30
54	32
56	33
58	35
60	37
62	39
64	41
66	42
68	44
70	46
72	48
74	49
76	51
78	53
80	55
82	56
84	58
86	60
88	62
90	63

BMI	Score

18	16
19	17
20	18
21	19
22	20
23	21
24	21
25	22
26	23
27	24
28	25
29	26
30	27
31	28
32	29
33	30
34	31
35	32
36	33
37	34
38	35
39	35

18	0

Polyps	Score
Yes	8
No	0

16	0
21	4
26	9
31	13
36	17
41	22
46	26
51	31
56	35

40	36
41	37
42	38
43	39
44	40
45	41
46	42
47	43
